# Mimivirus in Pneumonia Patients

**DOI:** 10.3201/eid1103.040538

**Published:** 2005-03

**Authors:** Bernard La Scola, Thomas J. Marrie, Jean-Pierre Auffray, Didier Raoult

**Affiliations:** * Université de la Méditerranée, Marseille, France; † University of Alberta, Edmonton, Alberta, Canada; ‡ Hôpital de Ste Marguerite, Marseille, France

**Keywords:** pneumonia, mimivirus, dispatch

## Abstract

Mimivirus, the largest virus known to date, is an amebal pathogen like *Legionella* spp. When mimivirus was used as an antigen in a migration inhibition factor assay, seroconversion was found in patients with both community- and hospital-acquired pneumonia. Mimivirus DNA was found in respiratory samples of patients with hospital-acquired pneumonia.

The causative agent of pneumonia, the leading cause of infection-related death throughout the world, is unknown in 20% to 50% of cases (*1*). Therefore, identifying new causative agents of both community- and hospital-acquired pneumonia is a major public health goal. Aquatic bacteria such as *Legionella* spp., *Pseudomonas* spp., *Stenotrophomonas* spp., *Burkholderia* spp., and *Acinetobacter* spp. colonize hospital water supplies. These bacteria, such as *L. pneumophila*, have been causally associated with both hospital- and community-acquired pneumonia. *Legionella* spp. and other bacteria are associated with free-living amebas in natural and hospital aquatic environments (*2*). Bacteria that resist phagocytic destruction by amebas and are found in aerosolized water are potential agents of pneumonia (*3*). Ameba-associated bacteria other than *L. pneumophila*, including other *Legionella* spp., new α-proteobacteria belonging to the Bradyrhizobiaceae (*Bosea* *massiliensis*) family, and members of the genus *Parachlamydia* might be implicated in hospital-acquired pneumonia (*4*–*6*).

In strict intraamebal bacteria, we found *Legionella*-like amebal pathogens (*7*), *Parachlamydia acanthamebae* (*8*), and a giant virus resembling gram-positive cocci that we named mimivirus (*9*). The mimivirus’s genome is bigger than that of *Mycoplasma* and genome sequencing is finished (*10*). Because we found antibodies against several ameba-associated bacteria in patients with community-acquired pneumonia and hospital-acquired pneumonia (*3*,*4*) in previous studies, we tested for antibodies to mimivirus by using a microimmunofluorescence assay on serum samples from patients with community-acquired pneumonia and hospital-acquired pneumonia. DNA of mimivirus was also found in the bronchoalveolar lavage specimens of patients with hospital-acquired pneumonia.

## The Study

We studied serum samples from 376 Canadian patients with community-acquired pneumonia (121 ambulatory and 255 hospitalized) and from 511 healthy control subjects. Extensive clinical data were available for 104 patients with community-acquired pneumonia. All of these samples were previously tested for other pneumonia agents (*4*). To prepare antigen for microimmunofluorescence study, mimivirus was grown in *Acanthameba polyphaga* strain Linc AP-1 in 75-cm^2^ cell culture flasks with peptone yeast extract glucose medium as previously described (*11*). After amebal lysis occurred, unlysed amebas were removed by low speed centrifugation at 100 g for 15 min. Mimivirus particles present in supernatant were centrifuged at 4,000 g for 30 min and washed 3 times in phosphate-buffered saline (PBS). The pellet obtained after the last washing was then resuspended in PBS at 2 mg/mL concentration of protein and used as antigen in microimmunofluorescence assay under previously described conditions (*5*). Evidence of serologic reaction to mimivirus was defined as 1) seroconversion from <1:50 to >1:100 between acute-phase and convalescent-phase serum samples or a 4-fold rise in antibody titer between acute-phase and convalescent-phase serum samples, or 2) a single or stable titer of >1:400. The cut-off titer for single serum was chosen to have <2.5% positive rate in control subjects. We also tested paired serum samples from 26 patients with intensive care unit (ICU)-acquired pneumonia for a 1-year period and 50 paired serum samples from patients in our institution to determine antibodies to *Rickettsia* spp. as controls.

To verify that antibodies against mimivirus in patients with pneumonia recognize mimivirus particles specifically, a serum sample of 1 of these patients was used to detect mimivirus particles as previously described (*12*). Two serum samples of patients who did not have detectable antibodies against mimivirus were used as controls. Grids were incubated briefly twice in incubation buffer (PBS with 0.2% bovine serum albumin) for 5 min, then for 15 min in lysine buffer (PBS with 0.05 mol lysin). Grids were washed twice in incubation buffer for 5 min, then incubated for 3 h at 37°C in patients’ diluted samples (diluted 1/1,000 in incubation buffer with 3% nonfat dry milk). Grids were washed 6 times for 5 min in incubation buffer, then incubated for 2 h at 37°C in goat antihuman immunoglobulin (IgG)-gold conjugate (Aurion Biovalley, Marne la Vallee, France) diluted 1/20 in incubation buffer with 3% nonfat dry milk. Grids were washed 6 times for 5 min in incubation buffer, then twice in PBS for 5 min. Grids were then immersed twice in gutaraldehyde (2% in PBS) for 5 min, rinsed 3 times in distilled water for 5 min, treated by R-GENT SE-EM (Aurion Biovalley) for 25 min, then rinsed 3 times in distilled water for 5 min before being stained with uranyl acetate before examination.

Genomic DNA of mimivirus was found in bronchoalveolar lavage specimens from patients in the ICU of the Ste. Marguerite Hospital, Marseilles, France; serologic reaction was studied in serum samples of the patients for 1 year. The study was retrospective; samples were tested anonymously from 12 to 18 months after sampling. Mimivirus was found in bronchoalveolar lavage specimens from 32 patients with ICU-acquired pneumonia and in specimens from 21 intubated control patients in ICU who did not have pneumonia (*5*). We designed 4 primer pairs chosen from the genome sequence of mimivirus. To avoid any contamination, we used a nested polymerase chain reaction (PCR), previously described as “suicide-PCR,” that incorporates 2 primer pairs used only once without positive control, followed by sequencing and comparing to the targeted sequence (*13*). DNA from patient and control BAL specimens, 3 water samples, and a suspension of *A. polyphaga* were extracted by using the QIAmp Tissue kit (QIAGEN GmbH, Hilden, Germany). DNA extracts from selected pathogens, including agents that are most commonly encountered in cases of hospital-acquired pneumonia (*14*), were tested: *Enterobacter* *aerogenes*, *Proteus mirabilis*, *Citrobacter freundii*, *Escherichia coli*, *Citrobacter koseri*, *Klebsiella pneumoniae*, *Enterobacter cloacae*, *Serratia marscescens*, *Haemophilus influenzae*, *Moraxella catarrhalis*, *Acinetobacter baumanii*, *Stenotrophomonas maltophila*, *Pseudomonas aeruginosa*, *Bacteroides fragilis*, *Prevotella intermedia*, *Streptococcus pneumoniae*, *Streptococcus oralis*, *Staphylococcus aureus*, *Staphylococcus epidermidis*, *Candida albicans*, CMV, HSV1, and adenovirus. Extracted DNA was used as template with primers BCFE (5′-TTATTGGTCCCAATGCTACTC-3′) and BCRE (5′-TAATTACCATACGCAATTCCTG-3′) as external primers and BCFI (5′-TGTCATTCCAAATGTTAACGAAAC-3′) and BCRI (5′-GCCATAGCATTTAGTCCGAAAG-3′) as internal primers. A minimum of 1 negative control was used for every 2 samples from patients with pneumonia; testing was conducted in a blinded manner.

## Conclusions

In the study of 511 healthy Canadian controls, 12 (2.3%) exhibited a substantial titer of antibodies to mimivirus. Patients with community-acquired pneumonia were positive more frequently than controls, as 36 (9.66%) were found positive (chi-square test, p < 0.01). Although the typical morphologic traits of mimivirus make its confusion with ameba organelles unlikely (Figure), positive serum samples tested on noninfected intact amebas suspended in PBS by using the same migration inhibition factor protocol did not show any reactivity. Immunoelectron microscopic examination showed that antibodies of positive patients recognize mature mimivirus particles specifically (Figure), whereas antibody fixation was not found in serum samples from 2 patients who were negative for mimivirus (data not shown). We compared selected features of 14 patients with community-acquired pneumonia who had serologic evidence of infection with mimivirus with those of 90 patients with community-acquired pneumonia who were seronegative for mimivirus (Table). Only hospitalization from a nursing home (3/14 vs. 3/90) and rehospitalization after discharge (6/14 vs. 16/90) were significantly associated with mimivirus antibodies (p < 0.05). Older age and diabetes mellitus were more common (both 6/14 versus 18/90) in patients with mimivirus antibodies but not significantly so (p = 0.07). In patients with community-acquired pneumonia, more frequent rehospitalization after discharge in patients with serologic evidence of mimivirus is likely explained by the poor efficacy of antimicrobial agents against viruses (*15*). Seropositive patients with community-acquired pneumonia were more likely to be admitted from a nursing home; this factor suggests that mimivirus is a particularly good candidate as an etiologic agent of pneumonia acquired in institutions, as is *L. pneumophila* (*16*). The seroprevalence of mimivirus was significantly higher than that of ameba–associated bacteria tested on the same samples (*4*).

**Figure:**
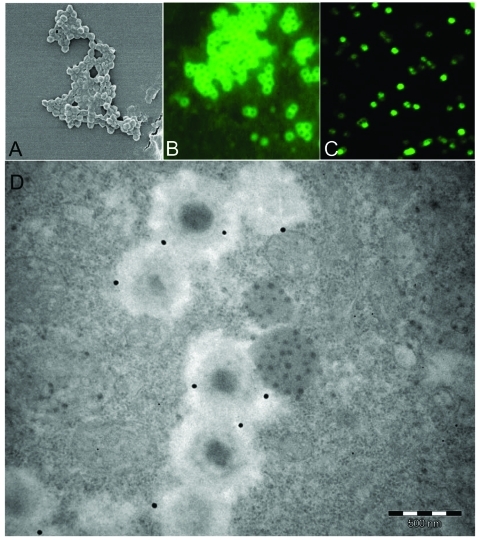
As observed by scanning electronic microscopy, mimivirus antigen (A) is recognized by antibodies in our microimmunofluorescence assay using conventional fluorescence microscope (B) and confocal microscope (C). Mature particles within amebas are also recognized by antibodies seen with transmission electronic microscopy immunogold technique (D) (mimivirus particle size 400 nm).

**Table:** Comparison of selected characteristics from patients with CAP who had serologic evidence of infection with Mimivirus with those of seronegative patients with CAP*

Characteristic	Positive (N = 14)	Negative (N = 90)	p
Age >80 y	6	18	0.07
Male	8	51	0.60
Length of hospital stay (d)	10.6	16.9	0.27
Days between onset and admission	6.9	5.2	0.77
Admission from nursing home	3	3	0.03
Retired	10	50	0.26
Smoked for >1 y	11	70	0.62
History of COPD	4	28	0.56
History of asthma	0	13	0.13
History of bronchiectasis	1	10	0.55
Diabetes mellitus	6	18	0.07
Hemodialysis	2	3	0.13
Rehospitalization after discharge	6	16	0.03
Death	2	2	0.08

*CAP, community-acquired pneumonia; COPD, chronic obstructive pulmonary disease.

Serologic evidence of infection was observed in 5 (19.2%) of 26 ICU patients. None of the 50 control patients was positive for mimivirus (p < 0.01). Mimivirus DNA was detected in bronchoalveolar lavage specimen from a 60-year-old comatose patient who had 2 episodes of hospital-acquired pneumonia during hospitalization in ICU. Mimivirus DNA was amplified from the second episode sample only. The sequenced amplified fragment was 100% homologous to the target DNA (GenBank accession no. AY026860). No serum sample was available from this patient. None of the DNA extracts from control microorganism showed a positive PCR reaction. The finding of mimivirus DNA in the bronchoalveolar lavage specimens from an ICU patient with nosocomial pneumonia confirms that mimivirus may reach the respiratory tract of these patients. Because of the procedure used for suicide PCR amplification, contamination appears highly unlikely. In this patient, however, we cannot distinguish colonization and infection, but this feature is common to most microorganisms isolated from respiratory samples.

This high rate of seroconversion observed in patients with pneumonia from our seroepidemiologic study suggests that community-acquired pneumonia and hospital-acquired pneumonia patients may have contact with mimivirus or a cross-reacting agent. As we do not report direct evidence of infection by mimivirus, these results have to be interpreted with caution. Viruses usually have a broad range of hosts, but the extraordinary size of the mimivirus genome (1.2 Mb), comparable to that of small bacteria such as *Mycoplasma* (*17*), suggests a possible adaptation to an extended range of hosts. We propose that mimivirus be tested as a possible novel human pathogen among ameba-resisting microorganisms. These results are preliminary, but raise the question of the pathogenic potential of the biggest identified virus to date. Mimivirus is an agent easy to cultivate and is freely available from our laboratory on request to researchers working on pneumonia who wish to introduce mimivirus antigen in serologic tests to confirm our results.

## References

[1] Marrie TJ, Durant H, Yates L. Community-acquired pneumonia requiring hospitalization: 5-year prospective study. Rev Infect Dis. 1989;11:586–99. 10.1093/clinids/11.4.586 2772465

[2] Barker J, Brown MRW. Trojan horses of the microbial world: protozoa and the survival of bacterial pathogens in the environment. Microbiology. 1994;140:1253–9. 10.1099/00221287-140-6-1253 8081490

[3] Greub G, Raoult D. Microorganisms resistant to free-living amebae. Clin Microbiol Rev. 2004;17:413–33. 10.1128/CMR.17.2.413-433.2004 15084508 PMC387402

[4] Marrie TJ, Raoult D, La Scola B, Birtles RJ, de Carolis E. *Legionella*-like and other amebal pathogens as agents of community-acquired pneumonia. Emerg Infect Dis. 2001;7:1026–9. 10.3201/eid0706.010619 11747734 PMC2631911

[5] La Scola B, Boyadjiev I, Greub G, Khamis A, Martin M, Raoult D. Amebae-associated bacteria from water are associated with culture negative ventilator-associated pneumonia. Emerg Infect Dis. 2003;9:815–21. 12890321 10.3201/eid0907.030065 PMC3023432

[6] Greub G, Berger P, Papazian L, Raoult D. *Parachlamydiaceae* as rare agents of pneumonia. Emerg Infect Dis. 2003;9:755–6. 12781026 10.3201/eid0906.020613 PMC3000139

[7] Birtles RJ, Rowbotham TJ, Raoult D, Harrison TG. Phylogenetic diversity of intra-amebal legionellae as revealed by 16S rRNA gene sequence comparison. Microbiology. 1996;142:3525–30. 10.1099/13500872-142-12-3525 9004515

[8] Birtles RJ, Rowbotham TJ, Storey C, Marrie TJ, Raoult D. *Chlamydia-*like obligate parasite of free-living amebae. Lancet. 1997;349:925–6. 10.1016/S0140-6736(05)62701-8 9093261

[9] La Scola B, Audic S, Robert C, Jungang L, de Lamballerie X, Drancourt M, A giant virus in amebae. Science. 2003;299:2033. 10.1126/science.1081867 12663918

[10] Raoult D, Audic S, Robert C, Abergel C, Renesto P, Ogata H. The 1.2-Mb genome sequence of Mimivirus. Science. 2004;306:1344–50. 10.1126/science.1101485 15486256

[11] Rowbotham TJ. Isolation of *Legionella pneumophila* from clinical specimens via amebae and the interaction of those and other isolates with amebae. J Clin Pathol. 1983;36:978–86. 10.1136/jcp.36.9.978 6350372 PMC498455

[12] Yu X, Brouqui P, Dumpler JS, Raoult D. Detection of *Ehrlichia chaffeensis* in human tissue by using species-specific monoclonal antibody. J Clin Microbiol. 1993;31:3284–8. 7508458 10.1128/jcm.31.12.3284-3288.1993 PMC266402

[13] Raoult D, Aboudharam G, Crubezy E, Larrouy G, Ludes B, Drancourt M. Molecular identification by “suicide PCR” of *Yersinia pestis* as the agent of Medieval Black Death. Proc 14. Natl Acad Sci U S A. 2000;97:12800–3. 10.1073/pnas.220225197 PMC18844 11058154

[14] Chastre J, Fagon JY. Ventilator-associated pneumonia. Am J Respir Crit Care Med. 2002;165:867–903. 11934711 10.1164/ajrccm.165.7.2105078

[15] Bartlett GJ, Dowell SF, Mandell LA, File TM, Musher DM, Fine MJ. Practice guidelines for the management of community-acquired pneumonia in adults. Clin Infect Dis. 2000;31:347–82. 10.1086/313954 10987697 PMC7109923

[16] El-Solh AA, Sikka P, Ramadan F, Davies J. Etiology of severe pneumonia in the very elderly. Am J Respir Crit Care Med. 2001;163:645–51. 11254518 10.1164/ajrccm.163.3.2005075

[17] Fraser CM, Gocayne JD, White O, Adams MD, Clayton PA, Fleishmann RD, The minimal gene complement of *Mycoplasma genitalium.* Science. 1995;270:397–403. 10.1126/science.270.5235.397 7569993

